# Neutrophil Elastase Defects in Congenital Neutropenia

**DOI:** 10.3389/fimmu.2021.653932

**Published:** 2021-04-22

**Authors:** Zuzanna Rydzynska, Bartlomiej Pawlik, Damian Krzyzanowski, Wojciech Mlynarski, Joanna Madzio

**Affiliations:** ^1^ Department of Pediatrics, Oncology and Hematology, Medical University of Lodz, Lodz, Poland; ^2^ Postgraduate School of Molecular Medicine, Medical University of Warsaw, Warsaw, Poland; ^3^ Laboratory of Epigenetics, Institute of Medical Biology, Polish Academy of Sciences, Lodz, Poland

**Keywords:** severe congenital neutropenia, cyclic neutropenia, neutrophil elastase, *ELANE* mutations, unfolded protein response, mistrafficking, mislocalization

## Abstract

Severe congenital neutropenia (SCN) is a rare hematological condition with heterogenous genetic background. Neutrophil elastase (NE) encoded by *ELANE* gene is mutated in over half of the SCN cases. The role of NE defects in myelocytes maturation arrest in bone marrow is widely investigated; however, the mechanism underlying this phenomenon has still remained unclear. In this review, we sum up the studies exploring mechanisms of neutrophil deficiency, biological role of NE in neutrophil and the effects of *ELANE* mutation and neutropenia pathogenesis. We also explain the hypotheses presented so far and summarize options of neutropenia therapy.

## Introduction

Functional neutrophils are the most abundant subpopulation of human leukocytes, representing the first line of the innate immune system defense against a broad spectrum of microorganisms (1). They exhibit an advanced antimicrobial mechanisms, from which we distinguish phagocytosis with, i.e. oxidative burst, release of nuclear material in the neutrophil extracellular traps (NETs) or degranulation of neutrophil granules (2).

Neutrophils are produced in hematopoietic cords inside venous sinuses in the bone marrow. Development of neutrophils, referred to granulopoiesis, begins with hematopoietic stem cells, (HSC) which may differentiate into common myeloid progenitor cells (3). These cells transform to granulocyte-monocyte progenitors, which in turn differentiate to neutrophils by intermediate stages of promyelocytes, myelocytes, metamyelocytes, band cells and segmented, polymorphonuclear cells (4). The main factor regulating both proliferation of neutrophil precursors and mature neutrophils release form the bone marrow is granulocyte colony-stimulating factor (G-CSF) (5). Disturbed production of neutrophils during hematopoiesis results in neutropenia in the peripheral blood, which leads to immunodeficiency. Among many causes of neutropenia, numerous genetic defects are described causative for congenital neutropenia (6). Mutations of neutrophil elastase gene (*ELANE)* are ones of the most commonly observed in patients suffering from congenital neutropenia (7). Nowadays, pathogenesis of these defects is still arguable and controversial, thus, it attracts a great scientific focus.

## Physiological Functions

Neutrophil elastase is one of the four serine proteases stored in the azurophil granules of neutrophils. It shows a relatively broad elastase specificity, preferring aliphatic amino acids, Ala, Val, and Ile, in the P1 position of substrates (8).

Serine proteases have been found to be a degradative non-specific enzymes as they are all able to cleave extracellular matrix proteins such as fibronectin, elastin, proteoglycans, collagens, the platelet IIb/IIIa receptor, cadherins and a wide range of plasma proteins (9–14). NE is also capable of degrading soluble proteins such as coagulation factors, complements immunoglobulins and many protease inhibitors (15). In addition to its role in degradation of extracellular matrix, neutrophil elastase functions as a negative regulator of the inflammation process. It degrades proinflammatory mediators such as IL-1β, TNF-α (16). Neutrophil elastase participates in the intracellular pathogen destruction with a potent antimicrobial activity against Gram-negative bacteria (17), spirochetes (18) and fungi (19). Bellaaouaj et al. generating strains of mice deficient in NE by targeted mutagenesis have shown that NE^-/-^ mice are more susceptible to sepsis and death due to infection with *Klebsiella pneumoniae* and *Escherichia coli*, both Gram-negative bacteria, as compared to wild type siblings (17). It has been proven that neutrophil elastase works directly through degradation of the outer membrane protein A (OmpA) located on the surface of *E. coli*. Additionally, *in vitro* incubation of NE with *E. coli* leads to a loss of bacterial membrane integrity or local weakening of the cell wall by degradation of Om protein A followed by osmotic lysis of bacteria (20). Alternatively, loss of wall integrity by OmpA cleavage may lead to NE entrance and intracellular protein degradation resulting in proteolysis and bacterial death. Hirche et al. have also shown that NE mediates innate host protection against *Pseudomonas aeruginosa* by degradation of the major outer membrane protein F (OmpF), a protein with important functions, including maintenance of membrane integrity, porin activity and sensing the host immune system activation (21).

Another direct function of human neutrophil elastase is the ability to cleave virulence factors such as flagellin, Gram-negative bacteria virulence factor with a strong proinflammatory activity on epithelial cells and other cell types (13). There is strong evidence that purified flagellin from *P. aeruginosa* is effectively degraded by neutrophil elastase and cathepsin G in the inhibitor dependent manner. What is more, López-Boado et al. have also shown in experimental conditions that none of the tested metalloproteinases (MMP-1, MMP-7 and MMP-8, which have a similar ability as NE) cleaved flagellin proving the role of NE (22).

Weinrauch et al. have shown that NE degrades *Shigella* virulence factors such as IpaA, IpaB and IpaC, as well as proteins secreted by *Salmonella* and *Yersinia* (SipA, SipB, SipC, HAPS and YopB, YopD, YopE, respectively) (23). Other *in vitro* studies have shown that NE plays a role in the neutrophil adhesion. The integrin Mac-1 binds to ligands such as fibrinogen and intercellular adhesion molecule 1 (ICAM-1) and mediates adhesion of neutrophils to the endothelial surface (24). Authors postulate that neutrophil elastase performs a proteolytic function by ICAM-1 cleavage in a manner dependent on the α1-antitrypsin (α1-AT) and *N*-methoxysuccinyl-Ala-Ala-Pro-Val-chloromethyl ketone (MSAAPVCK) – NE inhibitors (25).

Neutrophil proteases may also act indirectly through cleavage of serum proteins of the complement and coagulation systems to generate anti-microbial peptides. NE cleaves the central complement protein C3 to generate a peptide that imitates the natural C3a anaphylatoxin. Similarly to C3a, NE-derived C3 shows antimicrobial activity against *P. aeruginosa* and *E. faecalis* (26).

Apart from the intracellular degradation activity, neutrophil elastase demonstrates a broad spectrum of extracellular functions of which the best-known is connective tissue digestion in inflammation to clear the path for migrating neutrophils to reach sites of infection. It is capable of digesting many types of matrix proteins, including fibronectin, (denatured) collagen, proteoglycans, laminin and many others (27). Moreover, neutrophil elastase acts on cell‐surface ligands and receptors like CD14 (28) or TLR4 receptor and adhesion molecules like integrins. NE may activate TLR-4 receptors, which leads to the activation of Nuclear Factor κB (NFKB) and consequently, to the production of IL-8 (29, 30).

## *ELANE* Gene and NE Protein Structure

*ELANE* gene (also known as *ELA2*, *HLE*, *HNE*, *NE*, *SCN1*, according to OMIM 130130) encodes human neutrophil elastase (31). *ELANE* consists of five exons and six introns, and resides on chromosome 19 (19p13.3), the locus for several serine proteases (32, 33) *ELANE* is highly expressed at mRNA level in the bone marrow in promyelocytes and then it is downregulated with maturation of neutrophil.

**Figure 1:**
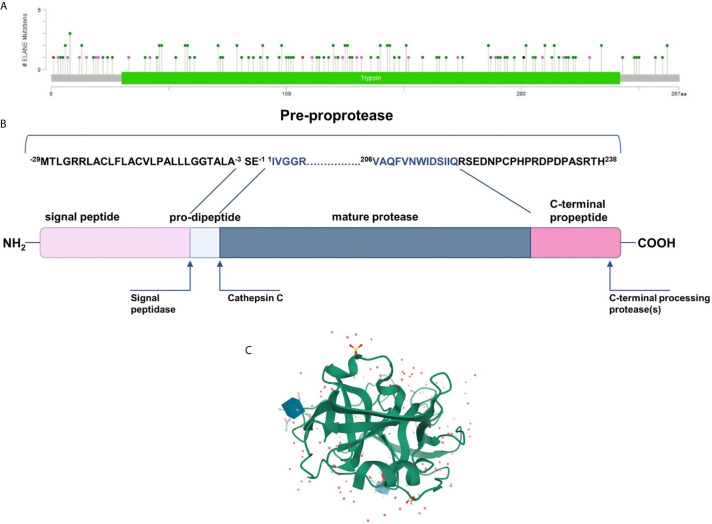
**(A)** Mutational spectrum of the neutrophil elastase (ClinVar database; RefSeq NM_001972.4) (34). The lolliplot reports mutations projected on the schematic Trypsin domain of the human NE protein. The lolliplot was produced using the MutationMapper software, freely available through the Cbioportal (https://www.cbioportal.org/mutation_mapper) (35, 36). In the scheme, mutations are reported as circles (green for missense mutations; black for duplication; brown for inframe deletions and insertions; violet for all the other types. **(B)** 267-residue preproprotein scheme and post-translational modification at both ends. **(C)** 3D structure of human neutrophil elastase 3Q76 (uncomplexed). Image created using Mol* from RCSB PDB (37, 38).

NE is synthesized as an inactive form of pro-pre-enzyme (zymogen) of 267 amino acids, containing N-terminal, single pro-dipeptide and a C-terminal pro-peptide. For neutrophil elastase to be active it has to undergo four consecutive post-translational protein modifications. First, 29 amino acid signal peptide is removed by a signal peptidase cleavage. Then, NE proform is glycosylated on asparagine residues at position 109 and 159. Further, N-terminal dipeptide – SerGlu is removed by cysteine protease – cathepsin C. Subsequently, the cleavage of dipeptide results in structural rearrangement of N-terminal region, which becomes inserted into the protein core. Finally, the C-terminal pro-peptide is removed, which altogether leads to formation of catalytically active enzyme comprising 218 amino acid residues and a mass of 29 to 34 kDa (EC 3.4.21.37) (32) (**Figure 1B**). The enzyme responsible for cleaving off the C-terminal pro-peptide has not been identified so far.

Mature neutrophil elastase, like all chymotrypsin-like serine proteases, forms a fold of two β-barrels, each made of six anti-parallel β-sheets connected through a linker segment, and a carboxyl-terminal α-helical domain (**Figure 1C**) (39). The active-site residues are located in a crevice between the two β-barrels. This single polypeptide chain has two glycosylation sites, it is stabilized by four disulfide bridges and has no lysine or tyrosine residues which, together with the surface distribution of the positively charged arginine restudies, make it extremely cationic. This asymmetric formation gives the protein a very high isoelectric point of about 10.5 (40).

## *ELANE* Mutations in Pathogenesis of Congenital Neutropenia

*ELANE-induced* neutropenia is not related to a NE deficit itself, but rather to a dysfunction of theisprotease. Heterozygous mutations identified in *ELANE* gene result in a SCN, which might be a life threatening condition, or a cyclic neutropenia (CyN) with moderate to mild clinical features. Pathogenesis of SCN and CyN is a subject of many studies and scientists are still not able to unequivocally explain how dysfunctions in NE lead to maturation arrest of granulocytic differentiation. The first reports have presented a theory that mutations in *ELANE* lead to dysregulated vesicular sorting and trafficking. Another, an equally popular theory, indicates the role of the unfolded protein response (UPR) system, which is responsible for stress response in the endoplasmic reticulum (ER) by improperly folded proteins. As there is a wide spectrum of *ELANE* mutations, over the years of research, other hypotheses for the pathogenesis of congenital neutropenia have been proposed.

### *ELANE* Mutations

*ELANE* mutation was first mentioned in the Horwitz report on neutrophil elastase as a cause for cyclic neutropenia, and the next year, the same team identified *ELANE* mutations in patients with SCN. The findings suggested that both forms are molecularly closer to each other than it was previously assumed (33, 41). The general pattern of mutations in the SCN versus CyN seems to be different, nevertheless, up to a dozen mutations overlap in both groups (42, 43). Based on genetic databases, a total of more than 230 *ELANE* mutations have been described so far (44), all the mutations mentioned in this article are according to RefSeq NM_001972.4 and their distribution is shown in **Figure 1A** (34). The vast majority of pathogenic defects are single amino acid substitutions, but there are also some frameshifts, nonsense, inframe indels as well as splice junction loss (42). The entire *ELANE* deletions are not described in the context of neutropenia, and the few cases such as chromosome 19p terminal deletion including this gene phenotypically do not show neutrophil count deficiency (45). Interestingly, humans (45) and mice (46) with *ELANE* biallelic loss of function defect produce functional neutrophils with normal complete blood counts. Moreover, knock-out of the mutant allele in hematopoietic stem cells derived from SCN patients restores neutrophils maturation (47). This excludes the NE haploinsufficiency as a pathomechanism of SCN, and it is likely that the cause of neutropenia is not the lack of neutrophil elastase itself, but protease malfunction.

To understand the role of NE in neutrophil dysfunction, distribution of mutations across *EL-ANE* structure seems to be more crucial than the type of defects. Although no clear link between genotype and phenotype has been described so far, the latest research substantiates nonsense-mediated decay (NMD) as the explanation of diversity in *ELANE* variants pathogenicity. Mutations introducing premature termination codons (PTCs) upstream of the 50^th^ nucleotide position in early exons of *ELANE* (1–4 exons) trigger NMD – the mechanism that eliminates defective mRNA transcripts and prevents truncated protein translations. In contrast, the mutation downstream of the 50^th^ nucleotide position within a late exon (4^th^ and 5^th^) of *ELANE* avoids NMD, thus, produces defective NE that impairs neutrophil maturation and manifests as severe neutropenia (48). It is consistent with previous observations in neutropenic patients, exons 4 and 5 are enriched in nonsense and frameshift mutations, which result in disruption of the disulphide bond domain in C-terminus of elastase that is essential for its correct intracellular localization (39, 43, 49). An example is p.Gly214Arg mutation that leads to NE mislocalization predominantly to the nuclear and plasma membranes, which accelerates apoptosis of differentiating cells and manifests itself as a severe neutropenia phenotype (50).

An alternative mechanism is described based on observation of the mutations in AUG initiation codon p.Met1Ile and p.Met1Val (c.3G>A and c.1A>G) and the noncoding Kozak sequence (c.-3A>T). Disruption of the start signal forces translation from internal methionine codons, thus, produces N-terminus truncated protein that cannot be targeted toward ER and is probably accumulated in the nucleus (51). Hypothetically, mutations in the signal peptide such as A25V may also be responsible for nuclear localization (43).

A negative synergistic effect on NE protein structure has been described based on the case of a double mutation of *ELANE*. The authors suggest that not only the range of the molecular damage but also synergistic effect may contribute to distinct pathomechanisms (52). Depending on various amino acids that could be substituted in Ala57 site, different neutropenia phenotypes can be manifested. Bioinformatics predictions have shown that a hydrophilic amino acid causes a clash with the side chain of adjacent amino acids, changes tertiary structure of neutrophil elastase and leads to its malfunction (53).

Opinions are divided as to the risk of malignant transformation; however, the most recent evidence indicates that the development of acute myeloid leukemia is increased in patients harboring *ELANE* mutant (42, 43, 54). Mutations such as p.Gly214Arg and p.Cys151Tyr are related to a poor response to G−CSF and a more severe phenotype, whereas variants p.Ser126Leu and p.Pro139Leu are associated with a good prognosis (42, 55, 56)

### Neutrophil Elastase Mistrafficking

**Figure 2:**
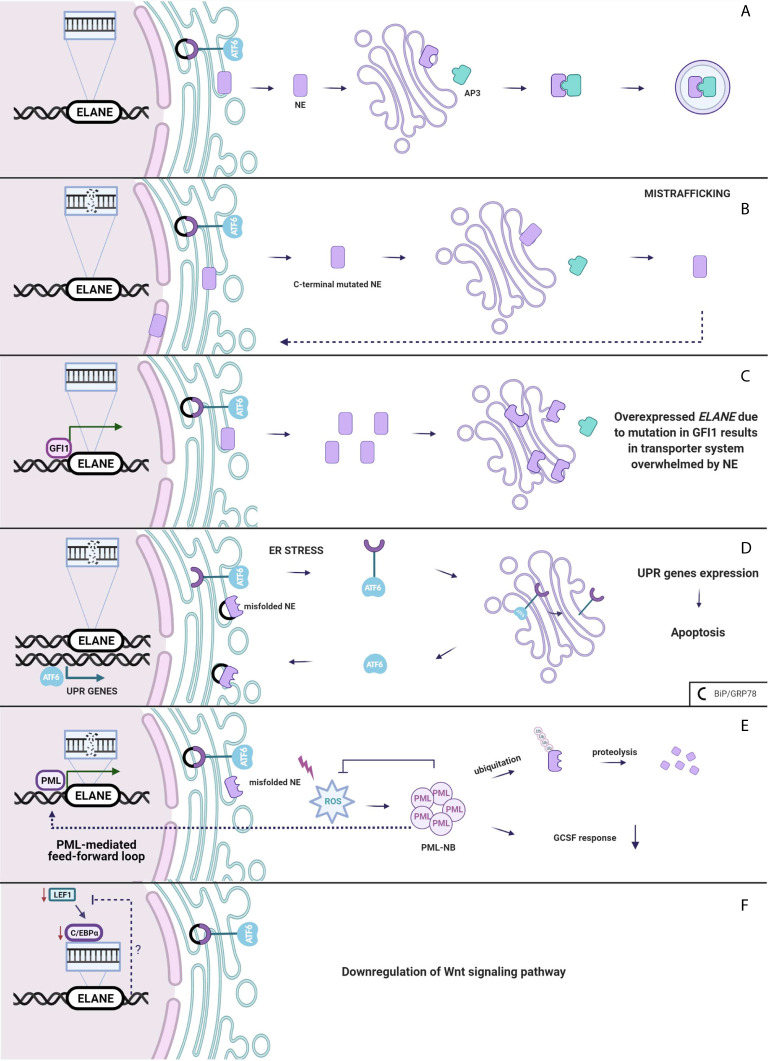
**(A)** Neutrophil elastase is trafficked from Golgi to the granules. **(B)** Mistrafficking. Mutation in *ELANE* results in intact C-terminal of NE disrupting an interaction between AP3 which mislocates it to the cytoplasm, plasma and nuclear membrane. **(C)** Overexpressed *ELANE* due to mutation in *GFI1* results in the transporter system overwhelmed by NE. **(D)** Unfolded protein response. Misfolded NE accumulation in ER leads to the ER stress and molecular chaperones upregulation and a subsequent induction of the UPR. **(E)** PML-mediated feed-forward loop. PML binds to *ELANE* promoter inducing misfolded NE expression thus increases ROS production and PML-NBs formation. **(F)** Either Lef-1 downregulates *ELANE* downstream or the Wnt pathway is downregulated through a negative feedback loop from mutated *ELANE*. Created with BioRender.com.

Neutrophil elastase belongs to the family of serine proteases located mainly in the azurophilic granules such as zymogens. Normally, the inactive serine protease undergoes post-translational modifications at the N-terminus and C-terminus. Although intact C-terminal does not affect enzymatic activity (57), its processing is crucial for recognition of NE by µ3a subunit of adaptor protein-3 (58, 59). AP-3 complex facilitates intracellular trafficking of NE from trans-Golgi network to the granule lumen (**Figure 2A**).

Absence of AP-3 significantly reduces the amount of neutrophil elastase in the granules, leading to SCN in humans (60) and CyN in dogs (58). Mutations in *ELANE* cause a disruption of the interaction between NE and AP-3 resulting in mistrafficking and excessive routing of the enzyme to the plasma membrane (61), and the nuclear membrane (50) (**Figure 2B**). Additionally Köllner et al. described accumulation of NE in cytoplasm of primary granules in cells derived from congenital neutropenia patients (62). However, not the lack of NE in granule itself leads to neutropenia but the incorrect location of mutant protease provokes elastase aberrant behavior resulting in abnormal neutrophil maturation.

This leads to a hypothesis that the mutant NE does not completely lose its proteolytic properties and, through mislocalization, it spontaneously degrades molecules that are essential for promyelocyte development. Mutated, though still active, NE present in the cytoplasm and cell membrane may defectively interact with potential substrates such as granulocyte colony stimulating factor receptor (G-CSF-R) (63) and Notch-family proteins (64), which regulate granulopoiesis. Intriguingly, according to the Garg et al. article, neutrophil elastase is inactive, but significantly sequesters expressions of hematopoietic transcription factors like Gfi1, Cebpd, Cebpe, and Spi1, cell surface receptors Csf3r and Gr1 and Mpo granule protein, which all are markers of granulocyte differentiation (65).

### *GFI1* Overexpression Leads to NE Mislocalization

Interestingly, growth factor independence 1 (GFI1) has been found to have an indirect impact on NE mistrafficking. *GFI1* encodes a nuclear transcription factor repressor GFI1, and it is widely expressed in the immune cells, playing a role in the development of hematopoietic cells (66). It has been found that Gfi1-deficient mice develop severe neutropenia and fail to produce mature granulocytes (67, 68). In normal conditions, GFI1 binds to the *ELANE* promoter, thus, repressing it. Depending on the mutation in *GFI1*, *ELANE* expression may either be increased or remain unchanged (69). Overexpression of neutrophil elastase leads to accumulation of the enzyme in multiple subcellular locations (70). Functional consequences of NE mislocalization are associated with impaired granulocytic differentiation (**Figure 2C**). NE is predominantly localized within granules but was also detected in the nucleus (71, 72). NE and GFI1 both were tested to bind to the nuclear protein PFAAP5, which silenced in HSC impaired myeloid differentiation. Thus, it is very likely that mutations of NE may influence the NE-PFAAP5-GFI1 interactions and result in abnormal modulations of transcription (73).

### Misfolded NE Triggers the Unfolded Protein Response

#### Canonical UPR

Neutrophil elastase, like most eukaryotic proteins, is transported to the endoplasmic reticulum, where it folds and matures. Protein folding is a process strictly controlled by genes encoding ER-resident chaperones. Excessive unfolded proteins accumulation in ER leads to ER stress and a subsequent induction of the regulatory pathway called the unfolded protein response. UPR can respond in three ways. Firstly, it diminishes cell protein synthesis. Secondly, it enhances transcriptional activation of chaperones, which helps in the proper folding of proteins. If the first two systems fail, ER-stress continues and homeostasis cannot be evoked and as a final response, a cell death pathway is induced (74). There is much evidence which proves that overwhelmed UPR system aims towards apoptosis and gives rise to diseases such as: hepatic fibrosis (75), neurodegenerative diseases (76) and cancer (77). Induction of apoptosis through the UPR demands an extremely high accumulation of misfolded proteins at the site of ER. In SCN, at the promyelocytic stage, when majority of granulocytes are arrested, mutated NE is highly expressed, thus, contributing to the cell death through apoptosis (78). Mutations in *ELANE* may cause neutrophil elastase misfolding that consequently aggregates in the promyelocyte cytoplasm and induces apoptosis through the UPR system (62). Indeed, diverse *ELANE* mutations are associated with upregulation of BiP/GRP78 – molecular chaperone, synthetized during UPR activation (79). Additionally, studies indicate that mutations causing cyclic neutropenia activate UPR in myeloid-derived cell lines to a lesser extent than *ELANE* mutations specific only for SCN (79, 80). In cyclic neutropenia, hematopoietic stem cell differentiation is not fully arrested, thus, some of the HSC are able to escape the UPR-induced ER stress, respond to G-CSF and generate neutrophils. However, most of the HSC carrying mutated NE will suffer from the UPR stress, thereby leading to cell death (81). Despite accelerated apoptosis, *in vitro* and *in vivo* studies (where one of the most severe NE mutation p.Gly214Arg was expressed) demonstrate that in SCN, neutrophil differentiation itself is not disrupted (50, 82). Due to the unsuccessful protein folding, the UPR system kills neutrophils before G-CSF induction, during the highest mutated NE expression, at the stage of promyelocyte (**Figure 2D**).

#### ROS-Induced Misfolded Protein Degradation

Abundant evidence indicates that UPR is one of the crucial mechanisms in SCN and CyN pathogenesis related to *ELANE* mutations. However, there are studies that support alternative scenarios. In a cellular model of HL-60, human promyeloblast cell line transfected with mutant NE, none of the studied UPR markers, neither BiP/GRP78 nor ATF6 were expressed (83). Likewise, in a similar *in vitro* model of granulopoiesis was impaired in *ELANE*-mutated cell line, albeit the UPR was not induced (51). In a study by Olofsen et al., there was no upregulation of aforementioned UPR-related genes in hematopoietic progenitor cells (HPC) derived from SCN patients with *ELANE* and *HAX1* mutations, but elevated levels of reactive oxygen species (ROS) were detected. However, only in HPC with *ELANE* mutations causing NE misfolding, levels of promyelocytic nuclear bodies (PML-NBs) were increased (84). PML-NBs are produced under excessive oxidative stress and target misfolded proteins for ubiquitination and further proteasomal degradation (85, 86). Moreover, misfolded NE activates ROS production that induces formation of PML-NBs. PML binds at the *ELANE* promoter and therefore, enhances the mutated *ELANE* transcription and misfolded NE expression that is described as a feed-forward mechanism. In addition, PML impairs the cell’s response to G-CSF. This leads to an alternative mechanism, where NE degradation is determined by the type of *ELANE* mutation and its influence on NE structure and folding, therefore, would proceed either through PML-NBs or by inducing the canonical UPR response triggered by ER-stress (**Figure 2E**) (84). However, as this is a newly described mechanism in terms of both pathogenesis and neoplastic transformation, more research on misfolded-related mutations and PML-NB needs to be performed.

### WNT Signaling in SCN Pathogenesis

In 2006, Skokowa et al., analyzed mRNA expression of various transcription factors in CD33+ cells derived from SCN patients or healthy individuals. The most significant difference between the two groups with respect to expression among transcription factors was detected in lymphoid enhancer-binding factor 1 (LEF-1). In promyelocytes of the patients with SCN, LEF-1 mRNA expression was 20-fold downregulated as compared to the healthy controls. LEF-1 was found to be a decisive transcription factor which mediates granulocyte differentiation into mature neutrophils (87). It belongs to the family of proteins included in the Wnt3a pathway. In the induced pluripotent stem cells (iPSCs) isolated from SCN patient with *ELANE* gene mutation, most of the genes related to the Wnt3a pathway including *LEF-1* and *C/EBP-α* were down-regulated. Moreover, addition of the Wnt3a protein to the cultured NE-mutated iPSCs resulted in a dose-dependent increase of mature neutrophils, which was comparable with the results obtained after addition of G-CSF (88). Disruptions in the Wnt3a pathway appear to represent another mechanism of SN development (**Figure 2F**).

## Clinical Features and Therapy of SCN With *ELANE* Mutations

Congenital neutropenia is usually diagnosed in very young children and is linked with recurrent infectious incidences including recurrent pneumonia, mucositis, gingivitis, skin abscesses or otitis media. These bacterial infiltrates might result in life-threatening conditions including sepsis. Mainly bacterial infections are present in congenital neutropenia. All of these occurring within the first months of life (89). In general, the clinical presentation of congenital neutropenia is mainly classified as cyclic neutropenia (CyN) and severe congenital neutropenia (SCN), depending on intermittent or continuous lack of neutrophil-granulocytes in the peripheral blood, which corresponds with the occurrence of infections typical for neutropenia.

There are no specific clinical descriptions, which are associated with mutations of the *ELANE* gene (43). The neutrophil elastase gene is the only one identified so far as causative for cyclic neutropenia. Carriers of *ELANE* mutation suffered from pure neutropenia as a cause of immunodeficiency. No other clinical features apart from immunodeficiency are presented. The distinction between cyclic and severe forms is firstly based on clinical features and serial absolute neutrophil counts (ANC) measurement (90). CyN has an estimated prevalence of one per million in the population and is characterized by neutrophil counts fluctuating with generally 21-day periodicity between nearly normal and 0.2 × 10^9^/l. Similar intervals of recurrence of clinical presentation as fever, skin and oropharyngeal infections are observed (41, 90, 91). However, exact time intervals may vary even between patients with the same *ELANE* mutation ((42). Interestingly, the same *ELANE* mutation present within the family might be associated with a clinical phenotype of CyN and severe congenital neutropenia suggesting that also other factors affect the clinical manifestation of the disease (92). SCN is estimated to be 3-4 per million births and is defined as a chronic reduction in the absolute number of neutrophils circulating in the blood below 0.5 × 10^9^/l. SCN is manifested by more severe infectious episodes as CyN. The majority of symptoms are managed with antibiotics, specific glucocorticoids, anti-inflammatory drugs; however, the patient outcome depends on the G-CSF response (89). The G-CSF response in CyN patients is revealed as a shortening of the period of the cycle length from 21 days to approximately 14 days and, thus, improves treatment outcome (81, 83). In the case of *ELANE*-mutated SCN, bone marrow aspiration always shows a hematopoietic arrest at the promyelocytic stage of immature neutrophil precursors (93).

### Contemporary Therapy of *ELANE* SCN With G-CSF or Hematopoietic Stem Cells Transplantation

#### G-CSF

Treatment of choice for SCN is the administration of granulocyte-colony stimulating factor, which increases the neutrophil number and improves survival and quality of life (94). Filgrastim, as a human, recombinant G-CSF, stimulates the bone marrow to produce and release granulocytes into the bloodstream, thus ≥10-fold increases the neutrophil and significantly reduces the severity of infections (5). Most of the patients with *ELANE*-related congenital neutropenia require exogenous G-CSF treatment. Only a few cases who have no history of severe infection might be subjected to antibiotic prophylaxis and wait-and-watch therapeutic strategy.

#### HSCT

In approximately 20% of SCN cases, the ANCs cannot be achieved in patients considered as non-responders, when G-CSF dosage is more than 50 µg/kg/24h, whilst the neutrophil count still remains at the level of <0.5x10^9^ cells per l (95). In such cases, an allogeneic hematopoietic stem cell transplantation (HSCT) should be considered (96). Nevertheless, the optimal time point for transplantation in patients not responding to G-CSF is still debatable (97). A recent report from the French cohort has revealed that not only G-CSF unresponding patients but also patients with a high dose G-CSF requirement (>15 μg/kg/day) have benefited from HSCT by reducing the risk of the MDS and/or acute myeloid leukemia (AML) transformation (98). Another critical indication for HSCT is a presence of a mutation in *CSF3R* or *RUNX1* genes within the bone marrow of patients with SCN, which significantly increases the risk of development of MDS/AML (99).

### MDS/AML Transformation

The patients with CyN have no increased risk of myelodysplasia or leukemic transformation and patients with SCN have. Patients suffering from SCN have approximately 10% increased risk of development of myelodysplastic syndrome (MDS) and/or acute myelogenous leukemia (89, 100, 101). Some data suggest that particular mutations in *ELANE* and a high dose of G-CSF are responsible for MDS/leukemia risk (42). About 70% of MDS/AML patients acquire nonsense mutations affecting the cytoplasmic domain of CSF3R (the G-CSF receptor). This might evade the proapoptotic activity within myeloid precursor cells, which become prone to accumulate further genetic defects leading to leukemia. Importantly, mutations in the *RUNX1* gene are by far the most frequent somatic secondary mutations in SCN-MDS/AML and preferentially occurred in *CSF3R* mutation clones. Additionally, mutations in *SUZ12*, *ASXL1* and *EP300* genes were also identified (102). Recent studies on the mice model have shown tertiary mutations in *CXXC4* gene suggesting that TET2 dysfunction is also involved in MDS/AML evolution in SCN (103).

**Figure 3:**
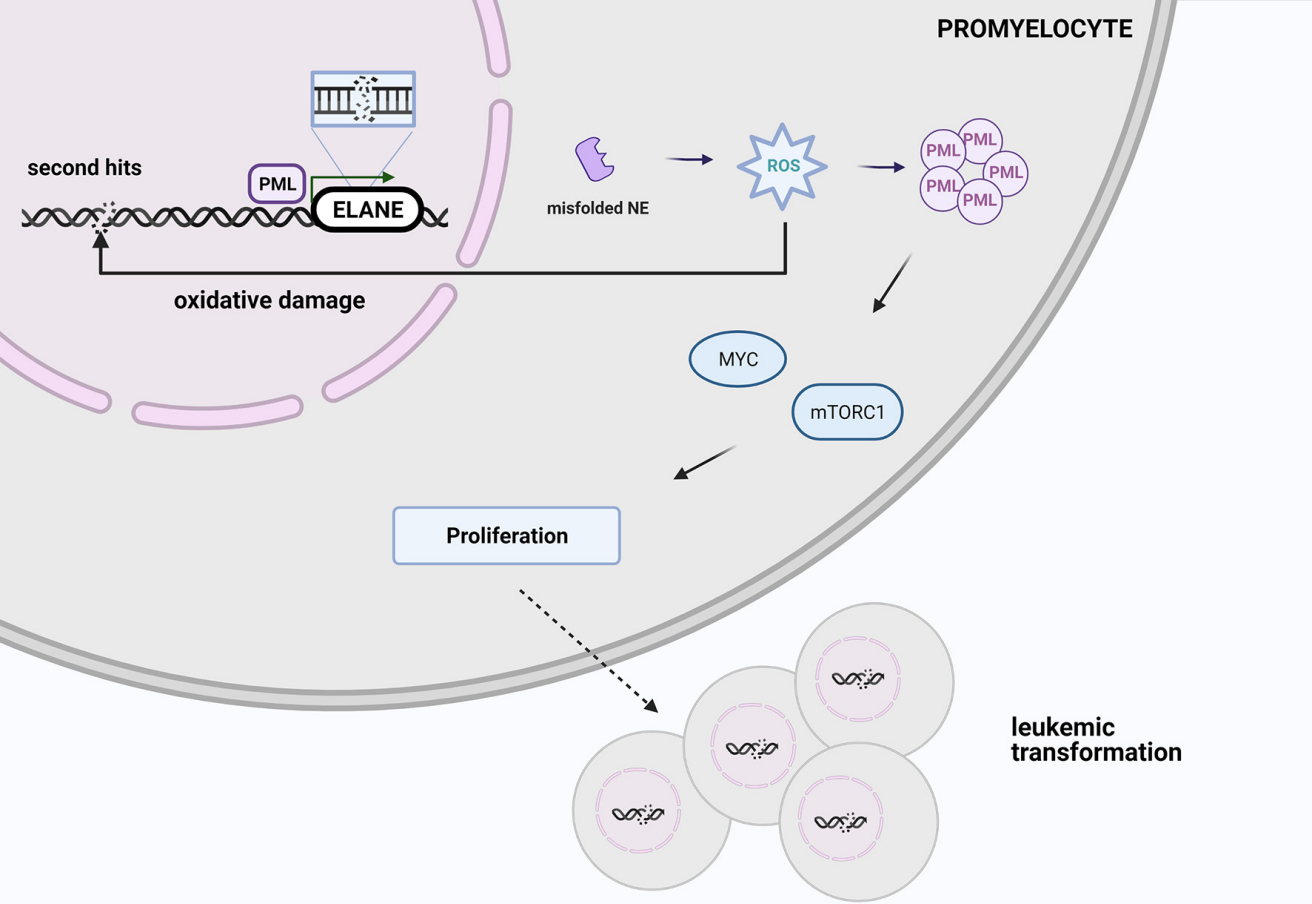
At the stage of promyelocyte the increased ROS level triggered by misfolded elastase leads to oxidative damage, which may be responsible for 2^nd^ hits and initiate malignant transformation. PML-NBs *via* MYC and mTOR signaling promotes malignant cell proliferation. Created with BioRender.com.

One possible explanation of leukemic risk among *ELANE* carriers is an increased ROS level induced by misfolded proteins leads to oxidative damage, which may be responsible for 2^nd^ hits and initiates malignant transformation, so AML or MDS development. PML-NBs *via* MYC and mTOR signaling induce metabolism and cell cycling that promotes malignant cell proliferation (**Figure 3**) (84). However, looking at the clinical perspective, since approximately 70% of SCN patients with AML harbor additional mutations in the *RUNX1* gene, patients with SCN who are positive for *CSF3R* and/or *RUNX1* mutation should be also transplanted to minimize the risk of MDS/AML occurrence.

### Future Therapeutic Perspectives

So far, no effective treatment has been developed for patients considered to be non-responders or resistant to G-CSF. Below, we present current efforts and potential implementation of novel methods based on NE inhibition, enhancing G-CSF functions or CRISPR/Cas9 *ELANE* gene edition that may help these patients in future studies. However, it should be noted, that the described methods do not yet have clinical application.

#### Neutrophil Elastase Inhibitors

To treat neutropenia an administration of endogenous recombinant or synthetic inhibitors of NE has been studied in preclinical or clinical settings. The first effort was focused on a small molecular weight compound, sivelestat sodium hydrate, which is a specific inhibitor of polymorphonuclear neutrophil elastase. Dokai et al. have shown decreasing apoptotic effects of neutrophil elastase after neutralization of its enzymatic activity by sivelestat in K562 and MEG-01 cell lines (104). In 2017, Makaryan et al. presented a report on four β-lactam-derived compounds, i.e. MK0339, L-910, L-538 and L-635. They also selected four *ELANE* mutations, p.Pro139Leu, p.Cys151Tyr, p.V174_C181del and p.Gly214Arg, on the model of patient-derived iPSC and HL60 cell line. One of the drugs, MK0339, showed not only improved survival of HL-60 cells and iPSc, but also their potential to differentiate to mature neutrophils. Pharmacokinetics studies of MK0339 have revealed that this drug achieves a plasma concentration of less than 0.5 mg/ml after an oral dosage of 10 mg/kg (83). Based on these preclinical studies, inhibition of neutrophil elastase might be a therapeutic option in future. However, some data also suggest that enzyme activity of elastase is not really linked with neutropenia, thus the further studies are needed in this field.

#### Nicotinamide

A small form of vitamin B3, nicotinamide (NAM), was previously described as responsible for the upregulation of G-CSF and G-CSF receptors expression, through NAD^+^/SIRT1 deacetylation in cellular models (105). Interestingly, to date, NAM at doses far above those recommended for vitamins is suggested to be an effective and safe therapeutic option for a wide spectrum of diseases and conditions, including neurological dysfunctions, psychological disorders and inflammatory diseases (106). In 2021, Deordieva et al. have shown, that combined therapy of G-CSF with NA may have a beneficial impact on increasing neutrophil count. Within 18 analyzed patients including 14 who harbored *ELANE* mutation, who received NA orally (20 mg/kg/day) and G-CSF (0.6-50.8 µg/kg/day), 78% showed a gradual increase of ANC after 3 months of therapy. Moreover, in half of the patients, treatment with NA reduced G-CSF effective dose, with complete NA replacement in one patient (107). These data have the strong and promising perspective for a reduction of G-CSF clinical effectiveness, especially in the case of patients with neoplastic predispositions.

#### CRISPR/Cas9 Gene Editing

Recent studies reveal a potential application of CRISPR/Cas9 mediated gene therapy in patients with *ELANE* mutated SCN. In 2019 Ritter et al. published a report on efficient correction of *ELANE* mutations in primary hematopoietic stem and progenitor cells (HSPCs). They selected four *ELANE* mutations in two exons (p.Ala57Val and p.Ala57Thr in exon 2 and p.Gly214Arg and p.Gly214Val in exon 5) that are hot spot mutations in non-responders to G-CSF. Using CRISPR/Cas 9 to edit these mutations and adeno-associated virus 6 (rAAV6) to deliver the template for repair the structure by homology directed repair (HDR), they successfully achieved an increasing number of mature neutrophils with NET formation and chemotaxis improved. At the same time, there were no changes in functionality of the produced cells (108). In 2020, another team supervised by Skokowa successfully restored neutrophil “maturation arrest” in SCN cells by complete knockout of the *ELANE* gene (*ELANE* KO). They observed granulocytic differentiation as well as phagocytic ability, reactive oxygen species production and chemotaxis by neutrophils without *ELANE* in SCN patient-derived iPSc, primary hematopoietic stem cells and HL-60 cell line with *ELANE* KO. It suggests an alternative method of SCN treatment using *ex vivo* CRISPR/Cas9 ribonucleoprotein mediated *ELANE* KO (47). An alternated approach to repair *ELANE* mutations was performed using two different single guide RNA (sgRNA) targeting both mutant allele and exon 4. Using this method, a group headed by Chu successfully repaired *ELANE* mutation in SCN patient-derived hematopoietic stem and progenitor cells together with restored neutrophil differentiation and normal elastase expression level (109).

Moreover, the group headed by Daniel E. Bauer confirmed, that complete loss of NE is not associated with SCN using a new approach of *ELANE* gene edition within early exons, which elicited nonsense-mediated decay (NMD). They found that -1 frame insertions or deletions that produce premature termination codons escaped from NMD and were responsible for neutrophil maturation arrest, whereas -2 frame indels are tied with translation repression and neutrophil maturation (48). Those findings may be useful in therapeutic gene editing of human HSPC that trigger NMD and can restore normal neutrophil production, at the same time lay the groundwork for a new and universal therapeutic strategy for *ELANE*-mutant SCN.

All the above-mentioned studies focus on promising data to further analyze a neutrophil elastase inhibitor or even gene editing by CRISPR/Cas9 method and may pave the way to the novel therapies for SCN.

## Conclusion

In light of the current literature and presented data on neutrophil elastase amount, localization, and activity there is no versatile model, which could explain how dysfunctions in NE lead to maturation arrest of granulocytic differentiation. Several hypotheses have emerged, the most popular of which indicates the role of dysregulated vesicular sorting and mistrafficking of mutated NE. The other theory points out the role of ER stress and unfolded protein response. In addition, genetic and epigenetic factors modulate these processes.

Although no clear genotype-phenotype correlation exists, the spectrum of mutations identified in neutropenic patients indicates that distribution of mutations across *ELANE* structure appears to be crucial. Recent studies have shown that *ELANE* whole gene deletion mutation does not cause neutropenia in humans and mice (45). Interestingly, *ELANE* knockout restores the granulopoiesis and increases differentiation into functional mature neutrophils *in vitro* (47). Therefore, we expect the neutrophil elastase gene to be a potential gene therapy target in patients with *ELANE* mutations, especially those who do not respond to G-CSF or who are at high risk of malignant transformation.

## Author Contributions

ZR initiated the review article, reviewed the literature and drew the figures. BP reviewed the literature and prepared drafts of the manuscript. DK evaluated the literature, drew the figures and edited drafts of the manuscript. WM contributed to the conception and provided input throughout. JM designed and edited drafts of the manuscript and finalized the manuscript for submission. All authors contributed to the article and approved the submitted version.

## Funding

The Foundation for Polish Science (FNP) TEAM NET Programme, POIR.04.04.00-00-1603/18. Project title: Fix Neutropenia (FIXNET): focusing on neutrophil proteases defects which serve as novel diagnostic and therapeutic options.

## Conflict of Interest

The authors declare that the research was conducted in the absence of any commercial or financial relationships that could be construed as a potential conflict of interest.

## References

[1] Borregaard N. Neutrophils, From Marrow to Microbes. Immunity (2010) 33:657–70. 10.1016/j.immuni.2010.11.011 21094463

[2] Rosales C. Neutrophil: A Cell With Many Roles in Inflammation or Several Cell Types? Front Physiol (2018) 9:1–17. 10.3389/fphys.2018.00113 29515456 PMC5826082

[3] Hong CW. Current Understanding in Neutrophil Differentiation and Heterogeneity. Immune Netw (2017) 17:298–306. 10.4110/in.2017.17.5.298 29093651 PMC5662779

[4] Cowland JB Borregaard N. Granulopoiesis and Granules of Human Neutrophils. Immunol Rev (2016) 273:11–28. 10.1111/imr.12440 27558325

[5] Mehta HM Malandra M Corey SJ. G-CSF and GM-CSF in Neutropenia. J Immunol (2015) 195:1341–9. 10.4049/jimmunol.1500861 PMC4741374 26254266

[6] Skokowa J Dale DC Touw IP Zeidler C Welte K. Severe Congenital Neutropenias. Nat Rev Dis Prim (2017) 3:17032. 10.1038/nrdp.2017.32 28593997 PMC5821468

[7] Arun AK Senthamizhselvi A Hemamalini S Edison ES Korula A Fouzia NA. Spectrum of Elane Mutations in Congenital Neutropenia: A Single-Centre Study in Patients of Indian Origin. J Clin Pathol (2018) 71:1046–50. 10.1136/jclinpath-2018-205235 30171085

[8] Fu Z Thorpe M Akula S Chahal G Hellman LT. Extended Cleavage Specificity of Human Neutrophil Elastase, Human Proteinase 3, and Their Distant Ortholog Clawed Frog Pr3-Three Elastases With Similar Primary But Different Extended Specificities and Stability. Front Immunol (2018) 9:2387. 10.3389/fimmu.2018.02387 30459762 PMC6232827

[9] Rao NV Wehner NG Marshall BC Gray WR Gray BH Hoidal JR. Characterization of Proteinase-3 (Pr-3), a Neutrophil Serine Proteinase: Structural and Functional Properties. J Biol Chem (1991) 266:9540–8. 10.1016/S0021-9258(18)92854-1 2033050

[10] Pipoly DJ Crouch EC. Degradation of Native Type IV Procollagen by Human Neutrophil Elastase. Implications for Leukocyte-Mediated Degradation of Basement Membranes. Biochemistry (1987) 26:5748–54. 10.1021/bi00392a025 3676285

[11] Heusinkveld LE Majumdar S Gao JL McDermott DH Murphy PM. Whim Syndrome: From Pathogenesis Towards Personalized Medicine and Cure. J Clin Immunol (2019) 39:532–56. 10.1007/s10875-019-00665-w PMC6698215 31313072

[12] Taylor JC Crawford IP Hugli TE. Limited Degradation of the Third Component (C3) of Human Complement by Human Leukocyte Elastase (Hle): Partial Characterization of C3 Fragments. Biochemistry (1977) 16:3390–6. 10.1021/bi00634a016 889803

[13] Anderssen T Halvorsen H Bajaj SP Osterud B. Human Leukocyte Elastase and Cathepsin G Inactivate Factor VII by Limited Proteolysis. Thrombosis Haemostasis (1993) 70(3):414–7. 10.1055/s-0038-1649596 8259540

[14] Si-Tahar M Pidard D Balloy V Moniatte M Kieffer N Van Dorsselaer A. Human Neutrophil Elastase Proteolytically Activates the Platelet Integrin A(Iib)B3 Through Cleavage of the Carboxyl Terminus of the A(Iib) Subunit Heavy Chain. Involvement in the Potentiation of Platelet Aggregation. J Biol Chem (1997) 272:11636–47. 10.1074/jbc.272.17.11636 9111081

[15] Epstein FH Weiss SJ. Tissue Destruction by Neutrophils. N Engl J Med (1989) 320:365–76. 10.1056/NEJM198902093200606 2536474

[16] Owen CA Campbell MA Boukedes SS Campbell EJ. Cytokines Regulate Membrane-Bound Leukocyte Elastase on Neutrophils: A Novel Mechanism for Effector Activity. Am J Physiol - Lung Cell Mol Physiol (1997) 272:L385–L93. 10.1152/ajplung.1997.272.3.L385 9124593

[17] Belaaouaj A Mccarthy R Baumann M Gao Z Ley TJ Abraham SN. Mice Lacking Neutrophil Elastase Reveal Impaired Host Defense Against Gram Negative Bacterial Sepsis. Nat Med (1998) 4:615–8. 10.1038/nm0598-615 9585238

[18] Garcia R Gusmani L Murgia R Guarnaccia C Cinco M Rottini G. Elastase is the Only Human Neutrophil Granule Protein That Alone is Responsible for in Vitro Killing of Borrelia Burgdorferi. Infect Immun (1998)66(4):1408–12. 10.1128/IAI.66.4.1408-1412 PMC108067 9529060

[19] Tkalcevic J Novelli M Phylactides M Iredale JP Segal AW Roes J. Impaired Immunity and Enhanced Resistance to Endotoxin in the Absence of Neutrophil Elastase and Cathepsin G. Immunity (2000) 12:201–10. 10.1016/S1074-7613(00)80173-9 10714686

[20] Belaaouaj AA Kwang SK Shapiro SD. Degradation of Outer Membrane Protein a in Escherichia Coli Killing by Neutrophil Elastase. Sci (80- ) (2000) 289:1185–7. 10.1126/science.289.5482.1185 10947984

[21] Hirche TO Benabid R Deslee G Gangloff S Achilefu S Guenounou M. Neutrophil Elastase Mediates Innate Host Protection Against Pseudomonas Aeruginosa. J Immunol (2008) 181:4945–54. 10.4049/jimmunol.181.7.4945 18802098

[22] López-Boado YS Espinola M Bahr S Belaaouaj A. Neutrophil Serine Proteinases Cleave Bacterial Flagellin, Abrogating Its Host Response-Inducing Activity. J Immunol (2004) 172:509–15. 10.4049/jimmunol.172.1.509 14688361

[23] Weinrauch Y Drujan D Shapiro SD Weiss J Zychlinsky A. Neutrophil Elastase Targets Virulence Factors of Enterobacteria. Nature (2002) 417:91–4. 10.1038/417091a 12018205

[24] Cai TQ Wright SD. Human Leukocyte Elastase is an Endogenous Ligand for the Integrin Cr3 (Cd11b/Cd18, Mac-1 A(M)B2) and Modulates Polymorphonuclear Leukocyte Adhesion. J Exp Med (1996) 184:1213–23. 10.1084/jem.184.4.1213 PMC2192826 8879192

[25] Champagne B Tremblay P Cantin A St Pierre Y. Proteolytic Cleavage of ICAM-1 by Human Neutrophil Elastase. J Immunol (1998) 161(11):6398–405. 9834131

[26] Nordahl EA Rydengård V Nyberg P Nitsche DP Mörgelin M Malmsten M. Activation of the Complement System Generates Antibacterial Peptides. Proc Natl Acad Sci USA (2004) 101:16879–84. 10.1073/pnas.0406678101 PMC534732 15550543

[27] Chua F Laurent GJ. Neutrophil elastase: mediator of extracellular matrix destruction and accumulation. Proc Am Thorac Soc. (2006) 3(5):424–7. 10.1513/pats.200603-078AW 16799086

[28] Le-Barillec K Si-Tahar M Balloy V Chignard M. Proteolysis of Monocyte CD14 by Human Leukocyte Elastase Inhibits Lipopolysaccharide-Mediated Cell Activation. J Clin Invest (1999) 103:1039–46. 10.1172/JCI5779 PMC408261 10194477

[29] Walsh DE Greene CM Carroll TP Taggart CC Gallagher PM O’Neill SJ. Interleukin-8 Up-Regulation by Neutrophil Elastase is Mediated by Myd88/IRAK/TRAF-6 in Human Bronchial Epithelium. J Biol Chem (2001) 276:35494–9. 10.1074/jbc.M103543200 11461907

[30] Devaney JM Greene CM Taggart CC Carroll TP O’Neill SJ McElvaney NG. Neutrophil Elastase Up-Regulates Interleukin-8 Via Toll-Like Receptor 4. FEBS Lett (2003) 544:129–32. 10.1016/S0014-5793(03)00482-4 12782302

[31] Fouret P Du Bois RM Bernaudin JF Takahashi H Ferrans VJ Crystal RG. Expression of the Neutrophil Elastase Gene During Human Bone Marrow Cell Differentiation. J Exp Med (1989) 169:833–45. 10.1084/jem.169.3.833 PMC2189275 2538548

[32] Takahashi H Nukiwa T Yoshimura K Quick CD States DJ Holmes MD. Structure of the Human Neutrophil Elastase Gene. J Biol Chem (1988) 263:14739–47. 10.1016/S0021-9258(18)68099-8 2902087

[33] Dale DC Person RE Bolyard AA Aprikyan AG Bos C Bonilla MA. Mutations in the Gene Encoding Neutrophil Elastase in Congenital and Cyclic Neutropenia. Blood (2000) 96:2317–22. 10.1182/blood.V96.7.2317.h8002317_2317_2322 11001877

[34] Landrum MJ Lee JM Benson M Brown GR Chao C Chitipiralla S. Clinvar: Improving Access to Variant Interpretations and Supporting Evidence. Nucleic Acids Res (2018) 46:D1062–7. 10.1093/nar/gkx1153 PMC5753237 29165669

[35] Gao J Aksoy BA Dogrusoz U Dresdner G Gross B Sumer SO. Integrative analysis of complex cancer genomics and clinical profiles using the cBioPortal. Sci Signal (2013) 6. 10.1126/scisignal.2004088 PMC4160307 23550210

[36] Cerami E Gao J Dogrusoz U Gross BE Sumer SO Aksoy BA. The cBio Cancer Genomics Portal: An open platform for exploring multidimensional cancer genomics data. Cancer Discov (2012) 2:401–4. 10.1158/2159-8290.CD-12-0095 PMC3956037 22588877

[37] Sehnal D Rose AS Koča J Burley SK Velankar S. Mol*: Towards a Common Library and Tools for Web Molecular Graphics. In: Workshop on Molecular Graphics and Visual Analysis of Molecular Data Molva ‘18 (2018). Goslar, DEU: Eurographics Association. p. 29–33. 10.2312/molva.20181103

[38] Hansen G Gielen-Haertwig H Reinemer P Schomburg D Harrenga A Niefind K. Unexpected active-site flexibility in the structure of human neutrophil elastase in complex with a new dihydropyrimidone inhibitor. J Mol Biol (2011) 409:681–91. 10.1016/j.jmb.2011.04.047 21549129

[39] Horwitz MS Corey SJ Grimes HL Tidwell T. Elane Mutations in Cyclic and Severe Congenital Neutropenia. Genetics and Pathophysiology. Hematol Oncol Clin North Am (2013) 27:19–41. 10.1016/j.hoc.2012.10.004 23351986 PMC3559001

[40] Korkmaz B Moreau T Gauthier F. Neutrophil Elastase, Proteinase 3 and Cathepsin G: Physicochemical Properties, Activity and Physiopathological Functions. Biochimie (2008) 90:227–42. 10.1016/j.biochi.2007.10.009 18021746

[41] Horwitz M Benson KF Person RE Aprikyan AG Dale DC. Mutations in ELA2, Encoding Neutrophil Elastase, Define a 21-Day Biological Clock in Cyclic Haematopoiesis. Nat Genet (1999) 23:433–6. 10.1038/70544 10581030

[42] Makaryan V Zeidler C Bolyard AA Skokowa J Rodger E Kelley ML. The Diversity of Mutations and Clinical Outcomes for ELANE-Associated Neutropenia. Curr Opin Hematol (2015) 22:3–11. 10.1097/MOH.0000000000000105 25427142 PMC4380169

[43] Germeshausen M Deerberg S Peter Y Reimer C Kratz CP Ballmaier M. The Spectrum of *ELANE* Mutations and Their Implications in Severe Congenital and Cyclic Neutropenia. Hum Mutat (2013) 34:905–14. 10.1002/humu.22308 23463630

[44] Stenson PD Ball EV Mort M Phillips AD Shiel JA Thomas NST. Human Gene Mutation Database (Hgmd®): 2003 Update. Hum Mutat (2003) 21:577–81. 10.1002/humu.10212 12754702

[45] Horwitz MS Laurino MY Keel SB. Normal Peripheral Blood Neutrophil Numbers Accompanying Elane Whole Gene Deletion Mutation. Blood Adv (2019) 3:2470–3. 10.1182/bloodadvances.2019000498 PMC6712528 31427279

[46] Martinod K Witsch T Farley K Gallant M Remold-O’Donnell E Wagner DD. Neutrophil Elastase-Deficient Mice Form Neutrophil Extracellular Traps in an Experimental Model of Deep Vein Thrombosis. J Thromb Haemost (2016) 14:551–8. 10.1111/jth.13239 PMC4785059 26712312

[47] Nasri M Ritter M Mir P Dannenmann B Aghaallaei N Amend D. Crispr/Cas9-Mediated ELANE Knockout Enables Neutrophilic Maturation of Primary Hematopoietic Stem and Progenitor Cells and Induced Pluripotent Stem Cells of Severe Congenital Neutropenia Patients. Haematologica (2020) 105:598–609. 10.3324/haematol.2019.221804 31248972 PMC7049355

[48] Rao S Yao Y Soares de Brito J Yao Q Shen AH Watkinson RE. Dissecting ELANE Neutropenia Pathogenicity by Human Hsc Gene Editing. Cell Stem Cell (2021) S1934–5909(20)30599–3. 10.1016/j.stem.2020.12.015 PMC8106646 33513358

[49] Nagy E Maquat LE. A Rule for Termination-Codon Position Within Intron-Containing Genes: When Nonsense Affects RNA Abundance. Trends Biochem Sci (1998) 23:198–9. 10.1016/S0968-0004(98)01208-0 9644970

[50] Massullo P Druhan LJ Bunnell BA Hunter MG Robinson JM Marsh CB. Aberrant Subcellular Targeting of the G185R Neutrophil Elastase Mutant Associated With Severe Congenital Neutropenia Induces Premature Apoptosis of Differentiating Promyelocytes. Blood (2005) 105:3397–404. 10.1182/blood-2004-07-2618 PMC1895019 15657182

[51] Tidwell T Wechsler J Nayak RC Trump L Salipante SJ Cheng JC. Neutropenia-Associated ELANE Mutations Disrupting Translation Initiation Produce Novel Neutrophil Elastase Isoforms. Blood (2014) 123:562–9. 10.1182/blood-2013-07-513242 PMC3901069 24184683

[52] Lanciotti M Caridi G Rosano C Pigullo S Lanza T Dufour C. Severe Congenital Neutropenia: A Negative Synergistic Effect of Multiple Mutations of ELANE (Ela2) Gene. Br J Haematol (2009) 146:578–80. 10.1111/j.1365-2141.2009.07787.x 19594744

[53] Park CH Park S Kim YJ Kim SH Kim HJ. Cyclic Neutropenia From a Novel Mutation Ala57Asp of ELANE: Phenotypic Variability in Neutropenia From Mutated Ala57 Residue. J Pediatr Hematol Oncol (2018) 42(4):e231–4. 10.1097/MPH.0000000000001353 31658467

[54] Rosenberg PS Alter BP Link DC Stein S Rodger E Bolyard AA. Neutrophil Elastase Mutations and Risk of Leukaemia in Severe Congenital Neutropenia. Br J Haematol (2008) 140(2):210–3. 10.1111/j.1365-2141.2007.06897.x PMC3143022 18028488

[55] Bellanné-Chantelot C Clauin S Leblanc T Cassinat B Rodrigues-Lima F Beaufils S. Mutations in the ELA2 Gene Correlate With More Severe Expression of Neutropenia: A Study of 81 Patients From the French Neutropenia Register. Blood (2004) 103:4119–25. 10.1182/blood-2003-10-3518 14962902

[56] Rosenberg PS Zeidler C Anna Bolyard A Alter BP Ann Bonilla M Boxer LA. Stable Long-Term Risk of Leukaemia in Patients With Severe Congenital Neutropenia Maintained on G-CSF Therapy. Br J Haematol (2010) 150:196–9. 10.1111/j.1365-2141.2010.08216.x PMC2906693 20456363

[57] Li FQ Horwitz M. Characterization of Mutant Neutrophil Elastase in Severe Congenital Neutropenia. J Biol Chem (2001) 276:14230–41. 10.1074/jbc.M010279200 11278653

[58] Benson KF Li FQ Person RE Albani D Duan Z Wechsler J. Mutations Associated With Neutropenia in Dogs and Humans Disrupt Intracellular Transport of Neutrophil Elastase. Nat Genet (2003) 35:90–6. 10.1038/ng1224 12897784

[59] Simpson F Peden AA Christopoulou L Robinson MS Darnell RB Robinson MS. Characterization of the Adaptor-Related Protein Complex, Ap-3. J Cell Biol (1997) 137(4):835–45. 10.1083/jcb.137.4.835 PMC2139840 9151686

[60] Dell’Angelica EC Shotelersuk V Aguilar RC Gahl WA Bonifacino JS. Altered Trafficking of Lysosomal Proteins in Hermansky-Pudlak Syndrome Due to Mutations in the B3a Subunit of the AP-3 Adaptor. Mol Cell (1999) 3:11–21. 10.1016/S1097-2765(00)80170-7 10024875

[61] Horwitz M Benson KF Duan Z Li F-Q Person RE. Hereditary Neutropenia: Dogs Explain Human Neutrophil Elastase Mutations. Trends Mol Med (2004) 10:163–70. 10.1016/j.molmed.2004.02.002 15059607

[62] Köllner I Sodeik B Schreek S Heyn H Von Neuhoff N Germeshausen M. Mutations in Neutrophil Elastase Causing Congenital Neutropenia Lead to Cytoplasmic Protein Accumulation and Induction of the Unfolded Protein Response. Blood (2006) 108:493–500. 10.1182/blood-2005-11-4689 16551967

[63] Hunter MG Druhan LJ Massullo PR Avalos BR. Proteolytic Cleavage of Granulocyte Colony-Stimulating Factor and Its Receptor by Neutrophil Elastase Induces Growth Inhibition and Decreased Cell Surface Expression of the Granulocyte Colony-Stimulating Factor Receptor. Am J Hematol (2003) 74:149–55. 10.1002/ajh.10434 14587040

[64] Duan Z Li F-Q Wechsler J Meade-White K Williams K Benson KF. Horwitz M. A Novel Notch Protein, N2n, Targeted by Neutrophil Elastase and Implicated in Hereditary Neutropenia. Mol Cell Biol (2004) 24:58–70. 10.1128/MCB.24.1.58-70.2004 14673143 PMC303357

[65] Garg B Mehta HM Wang B Kamel R Marshall S Corey SJ. Inducible Expression of a Disease-Associated Elane Mutation Impairs Granulocytic Differentiation, Without Eliciting an Unfolded Protein Response. J Biol Chem (2020) 295(21):7492–500. 10.1074/jbc.RA120.012366 PMC7247317 32299910

[66] Hock H Hamblen MJ Rooke HM Traver D Bronson RT Cameron S. Intrinsic Requirement for Zinc Finger Transcription Factor Gfi-1 in Neutrophil Differentiation. Immunity (2003) 18:109–20. 10.1016/S1074-7613(02)00501-0 12530980

[67] Karsunky H Zeng H Schmidt T Zevnik B Kluge R Schmid KW. Inflammatory Reactions and Severe Neutropenia in Mice Lacking the Transcriptional Repressor Gfi1. Nat Genet (2002) 30:295–300. 10.1038/ng831 11810106

[68] Geissler S Textor M Stumpp S Seitz S Lekaj A Brunk S. Loss of Murine Gfi1 Causes Neutropenia and Induces Osteoporosis Depending on the Pathogen Load and Systemic Inflammation. PloS One (2018) 13:1–21. 10.1371/journal.pone.0198510 PMC5991660 29879182

[69] Muench DE Olsson A Ferchen K Pham G Serafin RA Chutipongtanate S. Mouse Models of Neutropenia Reveal Progenitor-Stage-Specific Defects. Nature (2020) 582:109–14. 10.1038/s41586-020-2227-7 PMC8041154 32494068

[70] Person RE Li F Duan Z Benson KF Papadaki HA Eliopoulos G. Mutations in proto-oncogene GFI1 cause human neutropenia and target ELA2. Nat Genet (2003) 34(3):308–12. 10.1038/ng1170 PMC2832179 12778173

[71] Lane AA Ley TJ. Neutrophil Elastase Cleaves PML-Raralpha and is Important for the Development of Acute Promyelocytic Leukemia in Mice. Cell (2003) 115:305–18. 10.1016/S0092-8674(03)00852-3 14636558

[72] Armistead PM Wieder E Akande O Alatrash G Quintanilla K Liang S. Cyclic Neutropenia Associated With T Cell Immunity to Granulocyte Proteases and a Double De Novo Mutation in GFI1, a Transcriptional Regulator of ELANE. Br J Haematol (2010) 150:716–9. 10.1111/j.1365-2141.2010.08274.x PMC4112579 20560965

[73] Salipante SJ Rojas MEB Korkmaz B Duan Z Wechsler J Benson KF. Contributions to Neutropenia From PFAAP5 (N4BP2L2), a Novel Protein Mediating Transcriptional Repressor Cooperation Between Gfi1 and Neutrophil Elastase. Mol Cell Biol (2009) 29:4394–405. 10.1128/MCB.00596-09 PMC2725743 19506020

[74] Ron D Walter P. Signal Integration in the Endoplasmic Reticulum Unfolded Protein Response. Nat Rev Mol Cell Biol (2007) 8:519–29. 10.1038/nrm2199 17565364

[75] Maiers JL Malhi H. Endoplasmic Reticulum Stress in Metabolic Liver Diseases and Hepatic Fibrosis. Semin Liver Dis (2019) 39:235–48. 10.1055/s-0039-1681032 PMC6530577 30912096

[76] Shah SZA Zhao D Khan SH Yang L. Unfolded Protein Response Pathways in Neurodegenerative Diseases. J Mol Neurosci (2015) 57:529–37. 10.1007/s12031-015-0633-3 26304853

[77] Ojha R Amaravadi RK. Targeting the Unfolded Protein Response in Cancer. Pharmacol Res (2017) 120:258–66. 10.1016/j.phrs.2017.04.003 PMC5542584 28396092

[78] Pham CTN. Neutrophil Serine Proteases: Specific Regulators of Inflammation. Nat Rev Immunol (2006) 6:541–50. 10.1038/nri1841 16799473

[79] Grenda DS Murakami M Ghatak J Xia J Boxer LA Dale D. Mutations of the ELA2 Gene Found in Patients With Severe Congenital Neutropenia Induce the Unfolded Protein Response and Cellular Apoptosis. Blood (2007) 110:4179–87. 10.1182/blood-2006-11-057299 PMC2234798 17761833

[80] Nustede R Klimiankou M Klimenkova O Kuznetsova I Zeidler C Welte K. Elane Mutant-Specific Activation of Different Upr Pathways in Congenital Neutropenia. Br J Haematol (2016) 172:219–27. 10.1111/bjh.13823 26567890

[81] Mir P Klimiankou M Findik B Hähnel K Mellor-Heineke S Zeidler C. New Insights Into the Pathomechanism of Cyclic Neutropenia. Ann N Y Acad Sci (2020)1466(1):83–92. 10.1111/nyas.14309 32083314

[82] Wiesmeier M Gautam S Kirschnek S Häcker G. Characterisation of Neutropenia-Associated Neutrophil Elastase Mutations in a Murine Differentiation Model in Vitro and In Vivo. PloS One (2016) 11:1–20. 10.1371/journal.pone.0168055 PMC5152902 27942017

[83] Makaryan V Kelley ML Fletcher B Bolyard AA Aprikyan AA Dale DC. Elastase Inhibitors as Potential Therapies for ELANE -Associated Neutropenia. J Leukoc Biol (2017) 102:1143–51. 10.1189/jlb.5A1016-445R PMC5597518 28754797

[84] Olofsen PA Bosch DA Roovers O van Strien PMH de Looper HWJ Hoogenboezem RM. Pml-Controlled Responses in Severe Congenital Neutropenia With ELANE-Misfolding Mutations. Blood Adv (2021) 5:775–86. 10.1182/bloodadvances.2020003214 PMC7876869 33560392

[85] Guo L Giasson BI Glavis-Bloom A Brewer MD Shorter J Gitler AD. A Cellular System That Degrades Misfolded Proteins and Protects Against Neurodegeneration. Mol Cell (2014) 55:15–30. 10.1016/j.molcel.2014.04.030 24882209 PMC4445634

[86] Becker S Steinemann G Karle W Roos K Liem CH Muralikumar S. Stability of Smyd1 in Endothelial Cells is Controlled by PML-Dependent Sumoylation Upon Cytokine Stimulation. Biochem J (2021) 478:217–34. 10.1042/BCJ20200603 33241844

[87] Skokowa J Cario G Uenalan M Schambach A Germeshausen M Battmer K. LEF-1 is Crucial for Neutrophil Granulocytopoiesis and Its Expression is Severely Reduced in Congenital Neutropenia. Nat Med (2006) 12:1191–7. 10.1038/nm1474 17063141

[88] Hiramoto T Ebihara Y Mizoguchi Y Nakamura K Yamaguchi K Ueno K. Wnt3a Stimulates Maturation of Impaired Neutrophils Developed From Severe Congenital Neutropenia Patient-Derived Pluripotent Stem Cells. Proc Natl Acad Sci USA (2013) 110:3023–8. 10.1073/pnas.1217039110 PMC3581925 23382209

[89] Dale DC Cottle TE Fier CJ Bolyard AA Bonilla MA Boxer LA. Severe Chronic Neutropenia: Treatment and Follow-Up of Patients in the Severe Chronic Neutropenia International Registry. Am J Hematol (2003) 72:82–93. 10.1002/ajh.10255 12555210

[90] Dale DC Hammond WP IV. Cyclic Neutropenia: A Clinical Review. Blood Rev (1988) 2:178–85. 10.1016/0268-960X(88)90023-9 3052663

[91] Dale DC Bolyard AA Aprikyan A. Cyclic Neutropenia. Semin Hematol (2002) 39:89–94. 10.1053/shem.2002.31917 11957190

[92] Newburger PE Pindyck TN Zhu Z Bolyard AA Aprikyan AAG Dale DC. Cyclic Neutropenia and Severe Congenital Neutropenia in Patients With a Shared Elane Mutation and Paternal Haplotype: Evidence for Phenotype Determination by Modifying Genes. Pediatr Blood Cancer (2010) 55:314–7. 10.1002/pbc.22537 PMC2913300 20582973

[93] Liu Q Sundqvist M Li W Holdfeldt A Zhang L Björkman L. Functional Characteristics of Circulating Granulocytes in Severe Congenital Neutropenia Caused by ELANE Mutations. BMC Pediatr (2019) 19:189. 10.1186/s12887-019-1556-x 31176364 PMC6555947

[94] Bonilla MA Gillio AP Ruggeiro M Kernan NA Brochstein JA Abboud M. Effects of Recombinant Human Granulocyte Colony-Stimulating Factor on Neutropenia in Patients With Congenital Agranulocytosis. N Engl J Med (1989) 320:1574–80. 10.1056/NEJM198906153202402 2471075

[95] Dale DC Bonilla MA Davis MW Nakanishi AM Hammond WP Kurtzberg J. A Randomized Controlled Phase Iii Trial of Recombinant Human Granulocyte Colony-Stimulating Factor (Filgrastim) for Treatment of Severe Chronic Neutropenia. Blood (1993) 81:2496–502. 10.1182/blood.V81.10.2496.bloodjournal81102496 PMC4120868 8490166

[96] Ferry C Ouachée M Leblanc T Michel G Notz-Carrére A Tabrizi R. Hematopoietic Stem Cell Transplantation in Severe Congenital Neutropenia: Experience of the French Scn Register. Bone Marrow Transplant (2005) 35:45–50. 10.1038/sj.bmt.1704718 15489867

[97] Choi SW Boxer LA Pulsipher MA Roulston D Hutchinson RJ Yanik GA. Stem Cell Transplantation in Patients With Severe Congenital Neutropenia With Evidence of Leukemic Transformation. Bone Marrow Transplant (2005) 35:473–7. 10.1038/sj.bmt.1704813 15640815

[98] Rotulo GA Beaupain B Rialland F Paillard C Nachit O Galambrun C. Hsct May Lower Leukemia Risk in ELANE Neutropenia: A Before–After Study From the French Severe Congenital Neutropenia Registry. Bone Marrow Transplant (2020) 55(8):1614–22. 10.1038/s41409-020-0800-1 PMC7091645 31992846

[99] Skokowa J Steinemann D Katsman-Kuipers JE Zeidler C Klimenkova O Klimiankou M. Cooperativity of RUNX1 and CSF3R Mutations in Severe Congenital Neutropenia: A Unique Pathway in Myeloid Leukemogenesis. Blood (2014) 123:2229–37. 10.1182/blood-2013-11-538025 24523240

[100] Berliner N. Lessons From Congenital Neutropenia: 50 Years of Progress in Understanding Myelopoiesis. Blood (2008) 111:5427–32. 10.1182/blood-2007-10-077396 18544696

[101] Bejjani N Beaupain B Bertrand Y Bellanne-Chantelot C Donadieu J. How to Differentiate Congenital From Noncongenital Chronic Neutropenia At the First Medical Examination? Proposal of Score: A Pilot Study From the French Severe Chronic Neutropenia Registry. Pediatr Blood Cancer (2017) 64(12). 10.1002/pbc.26722 28727239

[102] Beekman R Valkhof MG Sanders MA Van Strien PMH Haanstra JR Broeders L. Sequential Gain of Mutations in Severe Congenital Neutropenia Progressing to Acute Myeloid Leukemia. Blood (2012) 119:5071–7. 10.1182/blood-2012-01-406116 22371884

[103] Olofsen PA Fatrai S van Strien PMH Obenauer JC de Looper HWJ Hoogenboezem RM. Malignant Transformation Involving CXXC4 Mutations Identified in a Leukemic Progression Model of Severe Congenital Neutropenia. Cell Rep Med (2020) 1(5):100074. 10.1016/j.xcrm.2020.100074 33205068 PMC7659587

[104] Dokai M Madoiwa S Yasumoto A Kashiwakura Y Ishiwata A Sakata A. Local Regulation of Neutrophil Elastase Activity by Endogenous A1-Antitrypsin in Lipopolysaccharide-Primed Hematological Cells. Thromb Res (2011) 128:283–92. 10.1016/j.thromres.2011.04.024 21624645

[105] Skokowa J Lan D Thakur BK Wang F Gupta K Cario G. NAMPT is Essential for the G-CSF-Induced Myeloid Differentiation Via a NAD+-Sirtuin-1-Dependent Pathway. Nat Med (2009) 15:151–8. 10.1038/nm.1913 19182797

[106] Hwang ES Song SB. Possible Adverse Effects of High-Dose Nicotinamide: Mechanisms and Safety Assessment. Biomolecules (2020) 10(5):687. 10.3390/biom10050687 PMC7277745 32365524

[107] Deordieva E Shvets O Voronin K Maschan A Welte K Skokowa J. Nicotinamide (Vitamin B3) Treatment Improves Response to G-CSF in Severe Congenital Neutropenia Patients. Br J Haematol (2021) 192(4):788–92. 10.1111/bjh.17313 33471934

[108] Ritter MU Secker B Klimiankou M Nasri M Aghaallaei N Dannenmann B. Efficient Correction of ELANE Mutations in Primary Hspcs of Severe Congenital Neutropenia Patients Using CRISPR/Cas9 and Ravv6 Hdr Repair Templates. Blood (2019) 134:1036–6. 10.1182/blood-2019-131708

[109] Tran NT Graf R Wulf-Goldenberg A Stecklum M Strauß G Kühn R. Crispr-Cas9-Mediated ELANE Mutation Correction in Hematopoietic Stem and Progenitor Cells to Treat Severe Congenital Neutropenia. Mol Ther (2020) 28:2621–34. 10.1016/j.ymthe.2020.08.004 PMC7704744 32822592

